# The moderating role of pre-adoptive reflective functioning in the association between early adversity and child difficulties after transnational adoption: a 4-year follow-up study

**DOI:** 10.1007/s00787-025-02782-x

**Published:** 2025-06-30

**Authors:** Simon Fiore, Nicole Vliegen, Bart Soenens, Patrick Luyten

**Affiliations:** 1 Faculty of Psychology and Educational Sciences, KU Leuven, Leuven, Belgium; 2 Department of Developmental, Personality and Social Psychology, Ghent University, Ghent, Belgium; 3 Research Department of Clinical, Educational and Health Psychology, University College London, London, UK

**Keywords:** Adoption, Reflective functioning, Pre-adoptive reflective functioning, Parental reflective functioning, Early adversity

## Abstract

Early adversity is associated with an increased risk for psychopathology and behavioral difficulties among transnational adoptees. Pre-adoptive reflective functioning may be an important buffer in this relationship. However, no studies have investigated this in adoptive families. Using longitudinal data from the Leuven Adoption Study (*N* = 48 participating families), this study investigated whether anthropometric proxies of early adversity (assessed in terms of child age, body mass index (BMI), weight for age, and length for age, based on parental reports of child weight and height at placement) predicted child difficulties assessed with the Child Behavior Checklist 4 years after child placement. The study also investigated the potential moderating role of adoptive parents’ pre-adoptive reflective functioning, as assessed by the Reflective Functioning Scale scored on the Adoption Expectations Interview. All associations were investigated controlling for the effects of child temperament, as measured by the short versions of the Infant Behavior Questionnaire or the Early Childhood Behavior Questionnaire. Results indicated that child age at placement and low BMI predicted socioemotional difficulties. Pre-adoptive reflective functioning among fathers, but not among mothers, buffered against the effect of early adversity on child socioemotional difficulties. Conversely, low paternal levels of reflective functioning were associated with greater effects of early adversity on socioemotional difficulties. This study identified fathers’ capacity for reflective functioning as a buffer against socioemotional difficulties. Implications for future research and practice, in terms of effective early interventions, are discussed.

**Supplementary Information:**

The online version contains supplementary material available at 10.1007/s00787-025-02782-x.

Early adversity includes exposure to adverse experiences such as neglect, abuse, and unstable caregiving during early childhood, which are likely to require significant psychological, behavioral, or neurobiological adaptations by children and can have a profound impact on their socioemotional development [1, 2]. This is particularly salient in the context of transnational adoption, where children often experience various forms of early adversity during the pre-adoptive period [3, 4]. Although comprehensive meta-analytic evidence suggests that most transnational adoptees do not significantly differ from their non-adopted peers, or catch up with them in later stages of development [4], there is considerable variability in adoptees’ socioemotional outcomes [5]. Specifically, experience of multiple adversities prior to adoption has been found to confer a heightened risk for long-term socioemotional difficulties. For example, one study found that transnational adoptees with multiple adversities prior to adoption were more likely to develop anxiety disorders (OR = 2.22; 95% CI 1.11–4.45), mood disorders (OR = 2.20; 95% CI:1.00–4.86), or substance abuse/dependence (OR = 3.81; 95% CI:1.62–8.98) in adulthood [5]. Hence, there is a need for research into factors underlying this variability.

Early adversity is widely recognized as a critical determinant of socioemotional outcomes, with numerous studies examining the role of its severity and duration in the adjustment of transnational adoptees, who are typically at increased risk for socioemotional difficulties, particularly when they have been exposed to higher levels of early adversity for prolonged periods [6–8]. However, progress in this area has been impeded by the limited availability of detailed information about children’s pre-adoptive experiences [5, 9]. In addition to pre-adoptive risk factors, research has increasingly focused on post-adoptive protective factors, such as sensitive parenting behaviors, which have been found to play an important role in supporting socioemotional adjustment [10, 11]. More recently, attention has shifted to the interaction between pre-adoptive risk factors and post-adoptive protective factors, highlighting their combined influence on socioemotional development in transnational adoptees [12, 13]. In this regard, parental reflective functioning [14, 15], that is, the capacity of parents to understand their own and their child’s behavior in terms of underlying mental states [16], has emerged as a key area of interest in research among at-risk children [14, 17]. However, despite its potential to buffer the effects of early adversity, there is a notable gap in studies exploring parents’ reflective functioning within the context of transnational adoption. To address this gap, the present study aims to investigate whether parents’ reflective functioning, assessed prior to adoption, moderates the relationship between early adversity and future socioemotional difficulties among their transnationally adopted children 4 years later. In what follows, research on early adversity in the context of transnational adoption and its role in socioemotional development will be discussed. Next, evidence regarding child age at placement and the potential use of anthropometric measures as proxies for early adversity will be reviewed. This will be followed by an overview of findings on the buffering role of parental reflective functioning. Finally, the study’s aims and hypotheses will be outlined.

## Early adversity in the context of transnational adoption

Studies have consistently indicated that transnationally adopted children often face significant adverse experiences prior to adoption. These experiences include neglect, abuse, inconsistent caregiving, and exposure to harsh conditions such as malnutrition and inadequate healthcare [2, 7, 19]. Although economic hardships in countries of origin and overburdened care systems often place all transnationally adopted children at increased risk for early adversity [18], the quality of pre-adoptive care and levels of adversity experienced vary widely [19]. For instance, while institutional care is typically associated with elevated rates of early adversity [20], the degree of adversity within such settings can vary substantially. Given that the majority of transnationally adopted children have lived for some time in institutional environments, such as orphanages or group homes, focusing solely on institutionalization status is insufficient for understanding the predictors of socioemotional adjustment over time.

A prominent area of research in transnational adoption has examined child age at placement as a predictor of socioemotional adjustment following adoption [1, 10]. Meta-analyses and systematic reviews have provided extensive evidence that older age at placement, typically defined as children older than 12 months at placement, is associated with increased rates of socioemotional maladjustment, including greater attachment insecurity [20], behavioral and emotional difficulties [21], and psychiatric disorders [22]. These findings are often interpreted through the lens of prolonged exposure to early adversity, as older children at placement are presumed to have experienced adversity for a longer duration. However, again, the heterogeneity in the quality of pre-adoptive care suggests that children adopted at the same age may have been exposed to vastly different levels of adversity. Consequently, relying solely on child age at placement as a proxy for early adversity may obscure other important factors influencing socioemotional outcomes. This underscores the need to incorporate additional predictors to better understand and address the development of socioemotional difficulties among transnational adoptees.

Given the frequent absence of detailed information about children’s early lives in the context of transnational adoption, anthropometric measures such as height, weight, and body mass index (BMI) at the time of adoption may serve as valid proxies for early adversity. Currently, a substantial body of evidence indicates that early adversity, including neglect in institutional facilities, is a significant risk factor for failure to thrive and growth delays in infancy and early childhood [23–25]. From an evolutionary perspective, theoretical frameworks suggest that prolonged exposure to physiological and social deprivation constrains growth as an adaptive mechanism to (temporarily) economize on somatic maintenance costs under harsh conditions [26]. Consistent with this notion, robust meta-analytic evidence demonstrates that transnationally adopted children often exhibit delayed growth at the time of arrival at their adoptive family followed by substantial recovery within the first years of placement in nurturing post-adoptive environments [7, 27]. For example, a longitudinal study of post-institutionalized transnational adoptees adopted at a mean age of 24 months indicated that these children showed significant growth delays in height and weight compared with their non-adopted peers immediately after adoption [28]. However, these differences were no longer significant by the time the children reached 4.5 years of age [28]. The severity of growth impairments also appears to be associated with the duration of children’s exposure to early adversity. Evidence suggests that post-institutionalized transnational adoptees who spent more than 6 months of their lives in institutional care exhibited significantly higher levels of stunted growth in terms of average height up to age 15. In contrast, no significant differences were observed among children adopted at younger ages [29]. However, few studies have examined the relationship between anthropometric measures at arrival and subsequent socioemotional adjustment, and even fewer have investigated how such proxies of early adversity interact with buffering factors in the post-adoptive environment.

## The moderating role of pre-adoptive reflective functioning

Parental reflective functioning is a relationship-specific form of reflective functioning that refers to the caregiver’s capacity to reflect upon their own mental states and their ability to perceive their child’s behavior as motivated by mental states [16]. A growing body of evidence indicates that parents’ capacity to understand the needs of their child plays a protective role in the socioemotional development of at-risk children. Studies have amply shown that parents’ reflective functioning can buffer the adverse effects of adversity experienced by parents in their own childhood on their children’s subsequent socioemotional adjustment [30], a process often discussed in the context of the intergenerational transmission of psychopathology [31, 32]. These protective effects are likely mediated by the ability of parents to engage sensitively with their child, guided by their understanding of the child’s behavior in terms of mental states [33–36]. Such sensitive parent–child interactions have been found to foster child attachment security and enhance emotion regulation [37], which may also mitigate the risk for socioemotional difficulties.

Given the potential protective role of parents’ reflective functioning in supporting at-risk children, it is hypothesized that this capacity may also benefit children with histories of early adversity, such as a substantial proportion of transnationally adopted children. Indirect support for this hypothesis comes from studies demonstrating the beneficial effects of interventions targeting parental reflective functioning on children’s socioemotional difficulties in the context of adoption [38] and foster care [39, 40]. However, no studies to date have directly and prospectively examined the buffering role of parents’ reflective functioning, assessed prior to adoption, in the association between early adversity and socioemotional difficulties among transnationally adopted children. Given the growing evidence for the stability of reflective functioning from pregnancy to 2 years postpartum [41–44], with correlations typically ranging from *r* =.52 to *r* =.86, the current study investigated whether this parental capacity, assessed prior to adoption, predicts socioemotional adjustment in adopted children. Addressing this gap is critical as it provides an opportunity to evaluate whether parents’ reflective functioning can serve as a pre-adoptive protective factor, offering insight into mechanisms that promote resilience in children who have faced early adversity. By investigating reflective functioning before adoption, this approach may also contribute to identifying characteristics of adoptive parents that support better socioemotional outcomes in their children, with implications for pre-adoptive preparation and post-adoption interventions.

## The present study

The first aim of this study was to examine whether child age at placement, as well as anthropometric measures—specifically BMI, weight for age, and length for age at placement (all based on parental reports)—can serve as proxies for early adversity, and prospectively predict socioemotional child difficulties 4 years post-adoption, as reported by their adoptive mothers and fathers. All analyses controlled for the potential effect of perceived child temperament on socioemotional adjustment, given the established association between temperament and socioemotional difficulties [45]. The second aim of this study was to investigate whether pre-adoptive reflective functioning of adoptive mothers and fathers, assessed prior to child placement, moderated the effects of early adversity on child socioemotional difficulties. In this regard, it was hypothesized that higher levels of pre-adoptive reflective functioning would buffer the impact of early adversity on total socioemotional difficulties, based on the assumption that higher levels of reflective functioning would enable adoptive parents to better understand and respond to their child’s needs in a sensitive manner, thereby fostering socioemotional development. In contrast, lower levels of pre-adoptive reflective functioning were expected to exacerbate the negative effects of early adversity, as lower levels of pre-adoptive reflective functioning may make it more difficult for parents to interpret their child’s behavior and may also confer a risk for parental insensitivity [33]. The third aim was to exploratively examine potential differences between adoptive mothers and fathers, as fathers are often underrepresented in research on adopted children [46].

## Methods

### Participants and procedure

The sample for this study consisted of 48 adoptive families from the Leuven Adoption Study (LAS), a multi-wave, multi-method, and multi-informant study on the development of transnationally adopted children and their parents in Flanders, Belgium [47]. The children comprised 34 boys (70.8%) and 14 girls (29.2%) who were a mean of 13.43 months old at placement (*SD* = 6.56, range = 4–30 months) and who were adopted from eight different countries: Ethiopia (27), South Africa (10), Kazakhstan (6), Burkina Faso (1), China (1), Nigeria (1), Sri Lanka (1), and Uganda (1).

All adoptive families were recruited through adoption agencies, social media and meetings of prospective adoptive parents. Couples who showed interest in participating in the LAS received a leaflet with further information about the study. If parents wished to participate, they met with a research assistant who provided further information, and gave written informed consent. At the time of participation, adoptive parents were a mean of 33.70 years old (*SD* = 3.59, range = 27–46) and all had gone through extensive psychosocial screening procedures carried out by social services of the Flemish agency for Public Health, Welfare and Family in order to receive admission to adopt by the central authority for adoption. All parents had Belgian nationality, were in a heterosexual relationship, had no biological children of their own, and had applied for transnational adoption for the first time. All families raised their child in the Dutch language, given that Dutch measures were used. Most adoptive parents were highly educated and were of medium to high socioeconomic status: 79.20% had participated in higher education, of whom 47.70% had obtained a bachelor’s degree and 37.50% a master’s degree. A table detailing the highest self-reported degree, broken down separately for adoptive mothers and fathers, is provided in the Supplementary Materials. To establish a homogeneous group of children, only families with preschoolers (children younger than 2.5 years at placement) were included at baseline.

The LAS collected data through interviews, behavioral observations, experimental tasks, and questionnaires assessing psychological constructs at multiple time points: prior to child placement, 2 weeks after placement, 6 months post-placement, and annually thereafter. For the current study, we used interview data collected before child placement, as well as questionnaire data gathered 2 weeks and 4 years after placement. Ethical approval for the study was obtained from the Social and Societal Ethics Committee of the University of Leuven (KU Leuven).

### Measures

#### Anthropometric measures

Child age, BMI, weight for age (WFA), and length for age (LFA), all measured at placement, were derived using data from the demographic questionnaires completed by adoptive parents 2 weeks after adoption. Child age at placement (in months) was calculated by subtracting the date of child arrival from the date of birth and was treated as a continuous variable. Anthropometric measures were computed using the WHO Anthro Software (http://www.who.int/childgrowth/software/en/). For all anthropometric measurements, exact *Z*-scores were computed based on the WHO standards, taking the child’s age and sex into account. The WHO standards are based on a pooled sample from six countries (Brazil, Ghana, India, Norway, Oman, and the USA) and are therefore suitable for use with a sample of transnationally adopted children.

#### Pre-adoptive reflective functioning before child placement

To assess adoptive parents’ reflective functioning before child placement, the Adoption Expectations Interview (AEI) [48], a semi-structured interview based on the Pregnancy Interview [49], was individually administered to adoptive mothers and fathers by trained research assistants. Previous studies have demonstrated good reliability and construct validity of reflective functioning scores derived from the AEI’s “demand questions” (which specifically probe for reflective functioning, e.g., “How did that make you feel?”) [48]. Initial evidence also supports the construct validity of these scores, as shown by significant correlations with self-report measures of mentalizing [48]. The AEI takes approximately 1.5 h to administer and consists of 20 questions, including (1) questions about the decision to adopt and the feelings related to the adoption procedure of both the interviewee and their partner, and the adoptive parent’s family and friends; (2) questions about current fantasies and feelings about the child, and what the parent thinks the child will need immediately after child placement; (3) questions about how prospective parents imagine themselves and their partner in their parental role and how this would be different from or similar to their own parents; and (4) questions about how they imagine their child’s future life will be [48]. For coding pre-adoptive reflective functioning, eight demand questions from the AEI were used [48], scored on the Reflective Functioning Scale [50], which ranges from −1 (negative) to 9 (exceptional or full). A final total score was then assigned to the interview on the same scale, ranging from −1 to 9. Scores from −1 to 3 were classified as negative to low reflective functioning, a score of 4 as borderline, and scores from 5 to 9 as average to high. Internal consistency of the demand items was excellent, with Cronbach’s α = 0.91. A more detailed description of the AEI, its question selection process, and its psychometric process has been previously published [48].

#### Child temperament

Child temperament was assessed 2 weeks after child placement with the short form of the Infant Behavior Questionnaire Revised (IBQ-R) [51] (for children aged <15 months) or the Early Childhood Behavior Questionnaire (ECBQ) [52] (for children aged 15–36 months), depending on the child’s age. Both questionnaires, which contain 37 and 36 items, respectively, assess three core dimensions of temperament—surgency, negative affectivity, and effortful control—consistent with the factor structure of the original scales [53]. Adoptive parents were asked to indicate on a seven-point Likert-type scale (never, seldom, less than half of the time, half of the time, more than half of the time, almost always, always, or not applicable) how often they had perceived specific child behavior in the past week (e.g., “When your baby wanted something, how often did he/she have tantrums (crying, screaming, face red, etc.) when he/she did not get what he/she wanted?” (IBQ-R), “When he/she asked you for something and you answered with ‘no’, how often did your child have a tantrum?” (ECBQ)). Scores for each dimension were calculated by averaging item scores that loaded on the respective dimensions [53, 54]. Finally, *Z*-scores were created for each questionnaire separately, using means and standard deviations of international samples published in previous studies [55–57]. Studies on the psychometric properties of the instruments showed satisfactory to good internal consistency and reliability [52, 55, 58]. Within our study sample, Cronbach’s alphas for surgency, negative affectivity, and effortful control were 0.55, 0.68, and 0.60, respectively, when measured with the IBQ-R, and 0.74, 0.75, and 0.62, respectively, when measured with the ECBQ.

#### Child socioemotional difficulties

The Child Behavior Checklist (CBCL) [59], one of the most widely used and validated questionnaires to assess socioemotional and behavioral difficulties among children, was completed by adoptive mothers and fathers separately. Depending on the child’s age 4 years after placement, the version for ages 1.5 to 5 (preschool; 99 items) or for ages 5 to 18 (school age; 118 items) was used. The CBCL has been shown to have strong psychometric properties and has been used in adoptive samples previously [13]. Parents were asked to indicate how often a statement with regard to their child was *not true* (0), *somewhat/sometimes true* (1), or *very true/often true* (3) during the past 6 months. The CBCL provides different scores; this study focused on total child difficulties and on internalizing and externalizing child difficulties. In order to analyze all scores together, *T*-scores were calculated based on the official US norms [59]. For the preschool version of the CBCL, Cronbach’s alphas were 0.94 for total difficulties, 0.80 for internalizing difficulties, and 0.91 for externalizing difficulties. For the school-age version, Cronbach’s alphas were 0.95 for total difficulties, 0.75 for internalizing difficulties, and 0.89 for externalizing difficulties.

### Statistical analyses

Preliminary analyses were conducted using SPSS Statistics 28.00. Descriptive statistics, including means and standard deviations, of the main study variables (child age, BMI, WFA, and LFA (all measured at placement), pre-adoptive reflective functioning, child socioemotional difficulties, and child temperament) and demographic variables were computed. Paired-samples *t*-tests were conducted to investigate possible differences between adoptive mothers and fathers on the study variables. Additionally, independent-samples *t*-tests were conducted to investigate whether there were differences between adopted boys and girls with regard to socioemotional difficulties and temperament. Next, Pearson correlations were calculated between the main study variables and demographic variables for mothers and fathers separately to investigate associations and to identify possible covariates. Finally, a Little’s Missing Completely at Random test (MCAR) was conducted to investigate possible patterns in missing data.

The main study hypotheses were investigated using Model 1 of Hayes’ PROCESS Macro for SPSS [60]. This macro automatically applies mean centering to continuous variables and uses a bootstrapping procedure (*n* = 5000) to examine the association between the predictor and the outcome at the low (16th percentile), middle (50th percentile), and high (85th percentile) levels of pre-adoptive reflective functioning, which was treated as a continuous variable. This analysis also relied on bootstrapping to generate bias-corrected confidence intervals for the simple slopes, with moderation effects considered significant when the confidence interval for a slope did not include zero. Each model included the main effects of child age at placement, BMI, and LFA, all measured at placement, as well as the main effect of pre-adoptive reflective functioning on socioemotional child difficulties. Additionally, each model examined one interaction effect between pre-adoptive reflective functioning and either child age at placement, BMI, or LFA on child difficulties. Child temperament (including the surgency, negative affectivity, and effortful control dimensions), child sex, and parental education (high school, bachelor’s degree, master’s degree) were included as covariates. Finally, in addition to analyzing the total child difficulties score, exploratory analyses were conducted to investigate whether the findings were consistent for internalizing and externalizing difficulties.

## Results

### Preliminary analyses

As can be found in Table 1, paired-samples *t*-tests indicated that adoptive mothers scored significantly higher than adoptive fathers in terms of pre-adoptive reflective functioning (*t*(47) = 2.75, *p* =.009). The Levene’s test showed no significant differences between mothers and fathers in variance for pre-adoptive reflective functioning (*F*(1,94) = 0.627, *p* =.430). Adoptive mothers and fathers did not differ significantly in their perceptions of socioemotional child difficulties or dimensions of child temperament (all *p*s > 0.05). Additionally, independent-samples *t*-tests showed that adopted boys and girls did not differ significantly in their age at placement, anthropometric measures, socioemotional difficulties, or temperament (all *p*s > 0.05). However, parents of adopted girls scored significantly higher on pre-adoptive reflective functioning compared with parents of adopted boys (*t*(94) =–0.53, *p* =.03). Finally, the Little’s MCAR test indicated that missing data were missing completely at random, χ^2^(22, *N* = 96) = 22.98, *p* =.40. Consequently, missing data were handled by using the expectation-maximization algorithm [61].

**Table 1:** Descriptive statistics of the main study variables for mothers and fathers separately

	Mothers	Fathers	t-value		*p*-value
*Demographic data*					
Parent age at child placement (years)	34.51 (3.44); [28.87–44.59]	35.81 (3.89); [28.69–47.47]	1.74	0.086^†^	
*Study variables*					
PA-RF	5.74 (1.03); [4–8]	5.27 (1.12); [3–8]	2.75	0.009^**^	
Child age at placement (months)	13.42 (6.60); [4–30]				
BMI *Z-*score	–0.10 (1.48); [–4.08–3.60]				
LFA *Z-*score	–2.04 (1.47) − 6.28–1.05]				
WFA *Z-*score	–1.29 (1.52) [–4.61–2.14]				
Total difficulties *T*-score	45.23 (9.53); [28–67]	45.42 (9.40); [29–70]	–0.209	0.836	
Internalizing difficulties *T*-score	43.96 (9.05); [29–63]	44.00 (8.90); [29–64]	–0.166	0.869	
Externalizing difficulties *T*-score	48.24 (10.45); [28–69]	48.26 (9.86); [28–75]	–0.228	0.821	
Surgency *Z*-score	–0.09 (1.02); [–2.34–2.40]	–0.09 (1.06); [–2.31–2.03]	0.009	0.992	
Negative affectivity *Z*-score	–0.19 (0.95); [–2.47–1.47]	–0.14 (0.90); [–2.59–2.02]	–0.406	0.687	
Effortful control *Z*-score	0.28 (0.89); [–1.72–2.26]	0.24 (0.93); [–1.42–2.35]	0.379	0.707	

Note. PA-RF = Pre-adoptive reflective functioning, BMI = Body mass index, LFA = Length for age, WFA = Weight for age, all measured at placement; ^†^*p* <.10. * *p* <.05. ** *p* <.01. *** *p* <.001; The mean, (SD) and [range] of each variable are provided. All adoptive parents were of middle to high socioeconomic status

Regarding the anthropometric measures, only a few children (8.9%; three boys and one girl) had a BMI indicative of being underweight (*Z*-score <–2). Nonetheless, there was substantial variation in BMI scores, indicating a spectrum of healthy and unhealthy weight. Moreover, 31.1% of the children had WFA or LFA *Z*-scores below −2. For each of these ratios, a score of −2 indicated a growth delay at the time of child placement, reflecting a ratio deviating by two or more standard deviations from the mean for children of the same age.

Regarding socioemotional difficulties, the US norms of the CBCL were used [59, 62], indicating that 13.5% of adoptive parents reported elevated externalizing difficulties, with 8.3% falling in the borderline range (*T*-scores between 60 and 63) and 7.2% in the clinical range (*T*-scores ≥64). For internalizing difficulties, 6.2% reported elevated difficulties, with 5.2% in the borderline range (*T*-scores between 60 and 63) and 1% in the clinical range (*T*-scores ≥64). A total of 8.3% of children exhibited elevated total socioemotional difficulties, with 5.1% in the borderline range (*T*-scores between 60 and 63) and 3% in the clinical range (*T*-scores ≥64). Levene’s tests showed no significant differences between mothers and fathers in variance for internalizing (*F*(1,75) = 0.008, *p* =.930), externalizing (*F*(1,75) = 0.147, *p* =.702), or total (*F*(1,75) = 0.060, *p* =.807) difficulties.

Pearson bivariate correlations were calculated separately for adoptive mothers and fathers and are presented in Table 2. Correlations between maternal and paternal reports of internalizing, externalizing, and total difficulties were *r* =.71 (*p* <.01), *r* =.78 (*p* <.01), and *r* =.76 (*p* <.01), respectively. To avoid multicollinearity due to the high correlation between BMI and WFA (*r* =.79), BMI was used in all subsequent models because it provides a comprehensive measure of both weight and height, making it a more holistic indicator of growth status compared with WFA. Outcomes for analyses using WFA as predictor are reported in the Supplementary Materials. Pre-adoptive reflective functioning was not significantly correlated with any other variables. However, it should be noted that correlation with other variables is not a requirement for a variable to serve as a moderator. With regard to potential covariates, maternal self-reported level of education was negatively associated with child age at placement. Therefore, while only maternal education showed a correlation, both maternal and paternal education levels were included as covariates to account for potential shared family-level influences in the analyses.

**Table 2:** *Pearson bivariate correlations for mothers and fathers separately*

		PA-RF	Child age	BMI	WFA	LFA	INT	EXT	TOT	SU	NA	EC	Education	Parent age
PA-RF			–0.09	–0.06	–0.02	0.03	–0.14	–0.11	–0.14	–0.16	–0.11	0.04	–0.04	0.19
Child age		–0.02		–0.03	0.10	0.23	0.38^*^	0.41^**^	0.48^**^	–0.46^***^	0.05	–0.52^**^	–0.35^**^	–0.01
BMI		0.17	–0.04		0.79^***^	0.13	0.05	–0.08	–0.06	0.03	0.17	0.01	0.26	–0.09
WFA		0.21	0.09	0.79^***^		0.71^***^	0.19	–0.06	0.02	–0.11	0.31^*^	–0.20	0.06	0.00
LFA		0.14	0.23	0.13	0.71^***^		0.28	0.03	0.15	–0.23	0.30^*^	0.35^*^	0.20	0.10
INT		–0.20	0.54^***^	–0.08	0.01	0.15		0.77^***^	0.91^***^	0.44^**^	0.24	–0.49^**^	0.13	–0.19
EXT		–0.05	0.56^***^	–0.10	–0.05	0.06	0.77^***^		0.94^***^	0.34^*^	0.16	–0.35^*^	0.11	0.03
TOT		–0.16	0.56^***^	–0.16	–0.06	0.11	0.89^***^	0.94^***^		–0.47^**^	0.16	–0.48^**^	0.07	–0.08
SU		–0.11	–0.40^**^	0.04	–0.01	–0.07	–0.21	–0.22	–0.21		0.14	0.66^***^	0.10	0.14
NA		–0.07	0.08	0.15	0.36^*^	0.42^**^	0.11	0.07	0.13	–0.01		–0.02	0.09	–0.05
EC		0.14	–0.51^***^	0.09	0.03	–0.08	–0.35^*^	–0.39^*^	–0.38^*^	0.32^*^	–0.16		0.00	–0.02
Education		0.27	–0.15	0.16	0.05	–0.09	–0.07	0.18	0.09	–0.00	–0.00	–0.29^*^		–0.15
Parent age		–0.02	0.16	–0.25	–0.17	–0.00	–0.01	0.19	0.13	0.29	–0.01	–0.14	–0.15	

Note. Correlations of adoptive mothers are presented above the diagonal, correlations of adoptive fathers are presented below the diagonal. ^†^*p* <.10. * *p* <.05. ** *p* <.01. *** *p* <.001. PA-RF = Pre-adoptive reflective functioning, BMI = Body mass index, WFA = Weight for age, LFA = Length for age, INT = Internalizing difficulties, EXT = Externalizing difficulties, TOT = Total difficulties, SU = Surgency, NA = Negative affectivity, EC = Effortful control

### Primary analyses

Results of the moderation analyses are presented in Tables 3, 4 and 5. Each model controls for the effects of other proxies used to assess early adversity, in addition to child temperament, child sex, and parent education.

**Table 3:** Child age at placement as predictor of total child socioemotional difficulties

	Mothers			Fathers		
	β (SE)	[95% CI]	*p*-value	β (SE)	[95% CI]	*p*-value
Main Effects						
CAAP ◊ Y	0.422 (0.161)	[0.097; 0.748]	0.012^*^	0.506 (0.160)	[0.186;0.834]	0.003^**^
PA-RF ◊ Y	–0.144 (0.145)	[–0.437; 0.149]	0.325	–0.197 (0.124)	[–0.448; 0.054]	0.121
Covariates						
BMI	–0.076 (0.089)	[–0.257; 0.105]	0.401	–0.100 (0.082)	[–0.267; 0.067]	0.233
LFA	0.050 (0.106)	[–0.164; 0.265]	0.638	0.032 (0.094)	[–0.159; 0.223]	0.739
SU	–0.212 (0.176)	[–0.569; 0.145]	0.237	–0.012 (0.119)	[–0.254; 0.230]	0.922
NA	0.121 (0.150)	[–0.183; 0.424]	0.426	0.120 (0.144)	[–0.172; 0.412]	0.411
EC	–0.167 (0.213)	[–0.599; 0.264]	0.436	–0.017 (0.177)	[–0.376; 0.343]	0.926
Child Sex	–0.076 (0.149)	[–0.377; 0.225]	0.610	–0.079 (0.126)	[–0.335; 0.177]	0.535
Parent Education	0.224 (0.166)	[–0.113; 0.561]	0.186	0.167 (0.131)	[–0.100; 0.433]	0.209
Interaction Effect:						
CAAP x PA-RF	–0.280 (0.161)	[–0.606; 0.048]	0.092†	–0.370 (0.142)	[–0.657;–0.082]	0.013^*^
Conditional Effects :						
Low PA-RF	–	–	–	1.018 (0.203)	[0.607; 1.429]	0.000^***^
Medium PA-RF	–	–	–	0.681 (0.149)	[0.378; 0.984]	0.000^***^
High PA-RF	–	–	–	0.176 (0.238)	[–0.307; 0.658]	0.465

Note. † *p* <.10. * *p* <.05. ** *p* <.01. *** *p* <.001. PA-RF = Pre-adoptive reflective functioning, CAAP = Child age at placement, BMI = Body mass index, LFA = Length for age, SU = Surgency, NA = Negative Affectivity, EC = Effortful Control. The values of PA-RF correspond with low (16th percentile), middle (50th percentile), and high (85th percentile) levels

**Table 4:** Child BMI at placement as predictor of total child socioemotional difficulties

	Mothers			Fathers		
	β (SE)	[95% CI]	*p*-value	β (SE)	[95% CI]	*p*-value
Main Effects						
BMI ◊ Y	–0.109 (0.093)	[–0.298; 0.080]	0.248	–0.088 (0.079)	[–0.247; 0.072]	0.275
PA-RF ◊ Y	–0.104 (0.151)	[–0.410; 0.201]	0.493	–0.067 (0.122)	[–0.247; 0.072]	0.596
Covariates						
CAAP	0.384 (0.168)	[0.045; 0.724]	0.028	0.648 (0.142)	[0.359; 0.936]	0.0001
LFA	0.009 (0.106)	[–0.206; 0.224]	0.933	0.042 (0.090)	[–0.140; 0.225]	0.642
SU	–0.233 (0.182)	[–0.602; 0.135]	0.208	–0.051 (0.114)	[–0.283; 0.180]	0.656
NA	0.146 (0.155)	[–0.168; 0.460]	0.352	0.094 (0.134)	[–0.185; 0.373]	0.498
EC	–0.157 (0.220)	[–0.603; 0.289]	0.481	0.140 (0.164)	[–0.192; 0.471]	0.398
Child Sex	–0.082 (0.155)	[–0.397; 0.234]	0.600	–0.213 (0.122)	[–0.461; 0.035]	0.090
Parent Education	0.226 (0.172)	[–0.123; 0.574]	0.197	0.271 (0.121)	[0.026; 0.517]	0.031
Interaction Effect:						
BMI x PA-RF	0.061 (0.091)	[–0.123; 0.245]	0.504	0.258 (0.078)	[0.101; 0.416]	0.002^**^
Conditional Effects :						
Low PA-RF	–	–	–	–0.442 (0.121)	[–0.688;–0.196]	0.001^***^
Medium PA-RF	–	–	–	–0.207 (0.081)	[–0.371;–0.043]	0.015^*^
High PA-RF	–	–	–	0.146 (0.114)	[–0.086; 0.378]	0.210

Note. † *p* <.10. * *p* <.05. ** *p* <.01. *** *p* <.001. PA-RF = Pre-adoptive reflective functioning, CAAP = Child age at placement, BMI = Body mass index, LFA = Length for age, SU = Surgency, NA = Negative Affectivity, EC = Effortful Control. The values of PA-RF correspond with low (16th percentile), middle (50th percentile), and high (85th percentile) levels

**Table 5:** Child LFA at placement as predictor of total child socioemotional difficulties

	Mothers			Fathers		
	β (SE)	[95% CI]	*p*-value	β (SE)	[95% CI]	*p*-value
Main Effects						
LFA ◊ Y	0.014 (0.122)	[–0.233; 0.260]	0.912	0.003 (0.102)	[–0.204; 0.209]	0.978
PA-RF ◊ Y	–0.162 (0.326)	[–0.823; 0.498]	0.622	–0.308 (0.234)	[–0.783; 0.167]	0.197
Covariates						
CAAP	0.413 (0.182)	[0.044; 0.781]	0.029	0.666 (0.161)	[0.338; 0.992]	0.0002
BMI	–0.098 (0.092)	[–0.284; 0.089]	0.296	–0.138 (0.088)	[–0.317; 0.041]	0.126
SU	–0.226 (0.185)	[–0.601; 0.149]	0.230	–0.012 (0.130)	[–0.275; 0.251]	0.926
NA	0.135 (0.156)	[–0.181; 0.452]	0.392	0.125 (0.157)	[–0.193; 0.442]	0.431
EC	–0.141 (0.222)	[–0.592; 0.309]	0.529	0.089 (0.186)	[–0.289; 0.466]	0.637
Child Sex	–0.064 (0.155)	[–0.378; 0.249]	0.679	–0.125 (0.135)	[–0.399; 0.145]	0.361
Parent Education	0.223 (0.174)	[–0.129; 0.576]	0.207	0.237 (0.139)	[–0.044; 0.518]	0.096
Interaction Effect:						
LFA x PA-RF	–0.023 (0.164)	[–0.356; 0.309]	0.887	–0.074 (0.106)	[–0.287; 0.140]	0.488
Conditional Effects :						
Low PA-RF	–	–	–	–	–	–
Medium PA-RF	–	–	–	–	–	–
High PA-RF	–	–	–	–	–	–

Note. † *p* <.10. * *p* <.05. ** *p* <.01. *** *p* <.001. PA-RF = pre-adoptive reflective functioning, CAAP = Child age at placement, BMI = Body mass index, LFA = Length for age, SU = Surgency, NA = Negative Affectivity, EC = Effortful Control. The values of PA-RF correspond with low (16th percentile), middle (50th percentile), and high (85th percentile) levels

#### Child age at placement

**Fig. 1:**
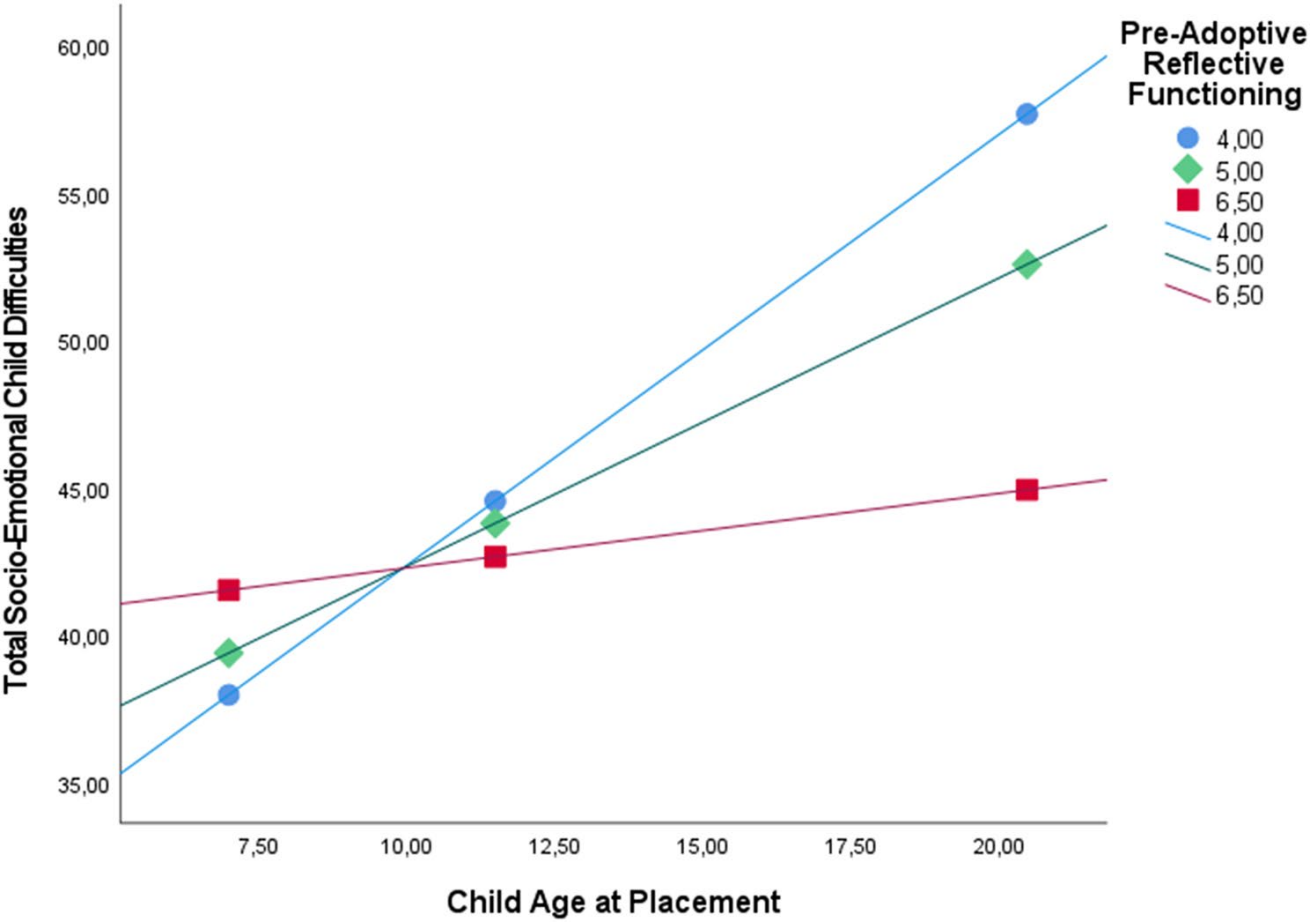
Paternal reflective functioning, child age at placement, and child total socioemotional difficulties 4 years after placement. The values of A pre-adoptive reflective functioning correspond with low (16th percentile), middle (50th percentile), and high (85th percentile) levels

Among adoptive fathers, the analyses indicated that the total model investigating child age at placement as main predictor explained a significant proportion of the variance in total socioemotional child difficulties as perceived by adoptive fathers (*R*^*2*^ = 0.54, *F*(10, 37) = 4.33, *p* <.001). Specifically, child age at placement significantly predicted total socioemotional child difficulties (*β* = 0.51, SE = 0.16, *p* =.003). Furthermore, the direct association between paternal pre-adoptive reflective functioning and total socioemotional child difficulties was not statistically significant (*β* =–0.20, SE = 0.12, *p* =.121). However, the interaction term between paternal pre-adoptive reflective functioning and child age at placement was statistically significant (*β* =–0.37, SE = 0.14, *p* =.013) and significantly moderated the association between child age at placement and total socioemotional child difficulties (*R*^2^ change = 0.08, *F*(1, 37) = 6.78, *p* =.013) (see Fig. 1). As Fig. 1 indicates, the association between child age at placement and child difficulties was most pronounced at the lowest level of pre-adoptive reflective functioning, less pronounced at intermediate levels, and non-significant at the highest level of pre-adoptive reflective functioning.

These findings were robust for internalizing child difficulties but not for externalizing difficulties. Specifically, child age at placement significantly predicted internalizing difficulties (*β* = 0.40, SE = 0.17, *p* =.025) and externalizing difficulties (*β* = 0.58, SE = 0.17, *p* =.001) as reported by adopted fathers. Furthermore, paternal pre-adoptive reflective functioning did not significantly predict internalizing difficulties (*β* =–0.23, SE = 0.14, *p* =.121) or externalizing difficulties (*β* =–0.11, SE = 0.14, *p* =.447). Finally, the interaction term between child age at placement and paternal pre-adoptive reflective functioning significantly predicted internalizing child difficulties (*β* =–0.34, SE = 0.154, *p* =.035), but did not significantly predict externalizing child difficulties (*β* =–0.22, SE = 0.15, *p* =.151).

Among adoptive mothers, the total model explained a significant proportion of the variance in total socioemotional child difficulties as perceived by adoptive mothers as well (*R*^*2*^ = 0.46, *F* (10, 37) = 3.19, *p* =.005). While child age at placement significantly predicted total socioemotional child difficulties as perceived by mothers (*β* = 0.42, SE = 0.16, *p* =.012), neither the direct effect of maternal pre-adoptive reflective functioning (*β* =–0.14, SE = 0.15, *p* = .325) nor the interaction effect of maternal pre-adoptive reflective functioning and child age at placement (*β* =–0.28, SE = 0.16, *p* =.092) significantly predicted total socioemotional child difficulties. The interaction of child age at placement and maternal pre-adoptive reflective functioning was not significant, but showed a trend toward statistical significance (*R*^2^ change = 0.04, *F*(1, 37) = 3, *p* =.092).

These findings were robust regarding externalizing child difficulties, as child age at placement significantly predicted externalizing difficulties (*β* = 0.47, SE = 0.18, *p* =.012). In contrast, the direct effect of child age at placement on internalizing difficulties was not statistically significant but showed a trend toward significance (*β* = 0.28, SE = 0.17, *p* =.113). Furthermore, maternal pre-adoptive reflective functioning did not significantly predict internalizing (*β* =–0.15, SE = 0.17, *p* =.371) or externalizing difficulties (*β* =–0.00, SE = 0.17, *p* =.978). Finally, the interaction between child age at placement and maternal pre-adoptive reflective functioning did not significantly predict internalizing difficulties (*β* =–0.21, SE = 0.17, *p* =.234) or externalizing difficulties (*β* =–0.28, SE = 0.18, *p* =.112), although there was a trend toward statistical significance in the prediction of externalizing child difficulties.

#### Children’s BMI at placement

**Fig. 2:**
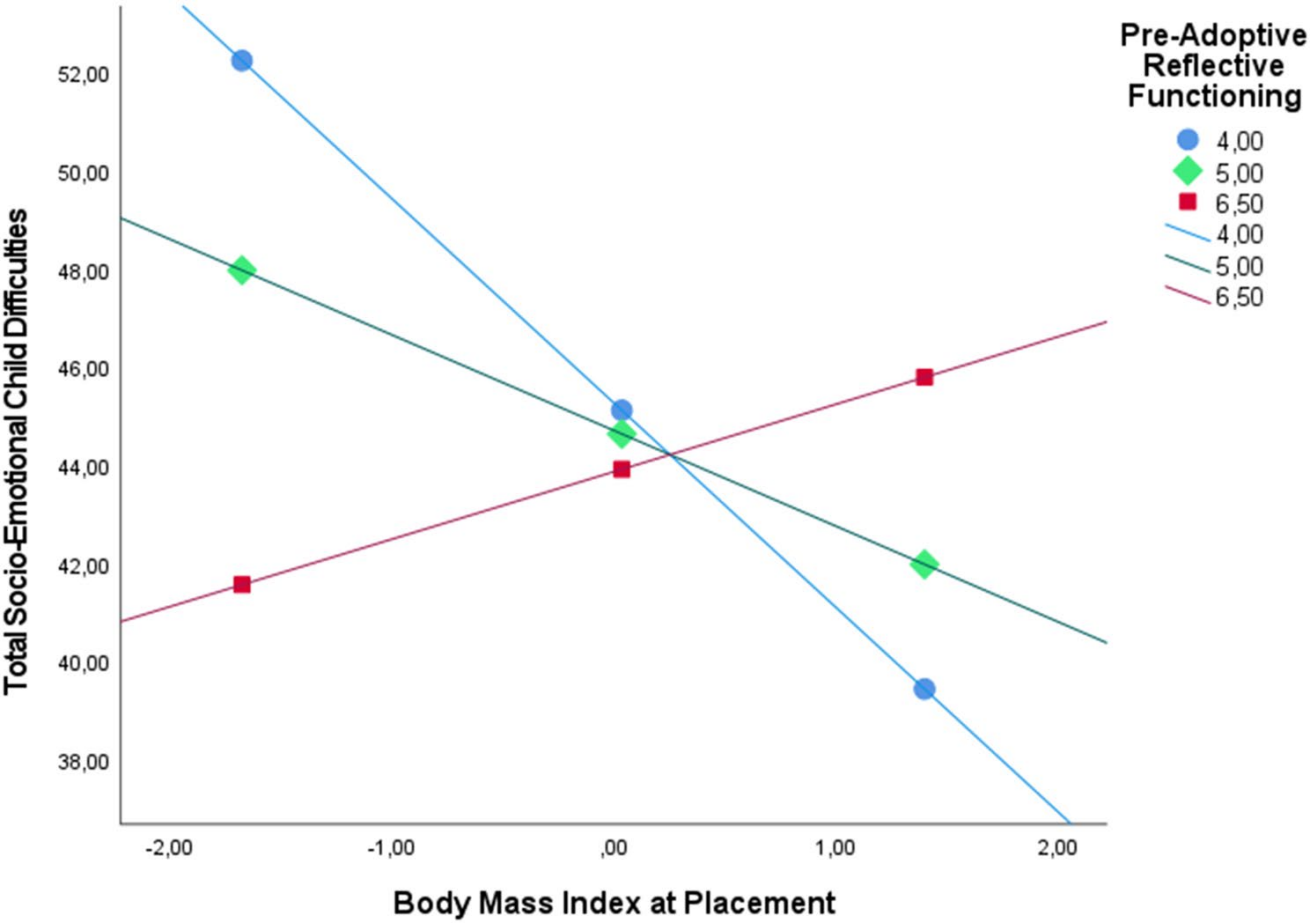
Paternal reflective functioning, child BMI, and child total socioemotional difficulties 4 years after placement. The values of pre-adoptive reflective functioning correspond to low (16th percentile), middle (50th percentile), and high (85th percentile) levels

Among adoptive fathers, the total model explained a significant proportion of the variance in total socioemotional child difficulties as perceived by adoptive fathers (*R*^*2*^ = 0.58, *F*(10, 37) = 5.11, *p* <.001). Neither child BMI at placement (*β* =–0.09, SE = 0.08, *p* =.275) nor paternal pre-adoptive reflective functioning (*β* =–0.07, SE = 0.12, *p* =.596) directly predicted total socioemotional difficulties. However, the interaction between child BMI at placement and paternal pre-adoptive reflective functioning did significantly predict total socioemotional child difficulties(*β* = 0.26, SE = 0.08, *p* =.002), and paternal pre-adoptive reflective functioning significantly moderated the association between child BMI at placement and total socioemotional child difficulties (*R*^2^ change = 0.13, *F*(1, 37) = 11.01, *p* =.002). As can be seen in Fig. 2, the associations with child BMI were most pronounced at the lowest level of pre-adoptive reflective functioning, less pronounced at intermediate levels, and non-significant at high levels.

These results were generally consistent for both internalizing and externalizing child difficulties, as child BMI at placement did not predict internalizing difficulties (*β* =–0.00, SE = 0.08, *p* =.953) or externalizing difficulties (*β* =–0.05, SE = 0.08, *p* =.540) as perceived by adoptive fathers. Furthermore, paternal pre-adoptive reflective functioning did not significantly predict internalizing difficulties (*β* =–0.06, SE = 0.13, *p* =.659) or externalizing difficulties (*β* = 0.02, SE = 0.13, *p* =.880). Similarly, consistent with the findings for total socioemotional child difficulties, the interaction between child BMI at placement and paternal pre-adoptive reflective functioning significantly predicted internalizing difficulties (*β* = 0.32, SE = 0.08, *p* <.001) and externalizing difficulties (*β* = 0.24, SE = 0.08, *p* =.003). However, a notable exception was observed for internalizing difficulties, where the significant moderation effect was found at both the lowest and highest levels of the moderator. In contrast, the moderation effect for total socioemotional difficulties was significant at the lowest and intermediate levels of the moderator.

Among adoptive mothers, the total model explained a significant proportion of the variance in total socioemotional child difficulties as perceived by adoptive mothers (*R*^*2*^ = 0.43, *F*(10, 37) = 2.76, *p* =.012). Furthermore, neither child BMI at placement (*β* =–0.11, SE = 0.09, *p* =.248) nor maternal pre-adoptive reflective functioning (*β* =–0.11, SE = 0.15, *p* =.493) significantly predicted total socioemotional child difficulties as perceived by mothers. Likewise, the interaction term between child BMI at placement and maternal pre-adoptive reflective functioning (*β* =–0.10, SE = 0.15, *p* =.493) was not statistically significant. Consequently, maternal pre-adoptive reflective functioning did not significantly moderate the association between child BMI at placement and total socioemotional child difficulties (*R*^2^ change = 0.01, *F*(1, 37) = 0.46, *p* =.504).

These results were robust for both internalizing and externalizing child difficulties, as child BMI at placement did not predict internalizing difficulties (*β* =–0.05, SE = 0.09, *p* =.618) or externalizing difficulties (*β* =–0.12, SE = 0.10, *p* =.254). Furthermore, maternal pre-adoptive reflective functioning did not directly predict internalizing (*β* =–0.11, SE = 0.17, *p* =.533) or externalizing difficulties (*β* = 0.03, SE = 0.18, *p* =.848). Finally, consistent with the findings for total socioemotional child difficulties, the interaction between child BMI at placement and maternal pre-adoptive reflective functioning did not significantly predict externalizing difficulties (*β* = 0.06, SE = 0.10, *p* =.576). However, the effect showed a trend toward significance for internalizing difficulties (*β* = 0.14, SE = 0.09, *p* =.135).

#### Children’s LFA at placement

Among adoptive fathers, the total model explained a significant proportion of the variance in total socioemotional child difficulties as perceived by adoptive fathers (*R*^*2*^ = 0.46, *F*(10, 37) = 3.18, *p* =.005). Neither child LFA at placement (*β* = 0.00, SE = 0.10, *p* =.978) nor paternal pre-adoptive reflective functioning (*β* =–0.31, SE = 0.23, *p* =.197) directly predicted total socioemotional difficulties. Furthermore, the interaction between child LFA at placement and paternal pre-adoptive reflective functioning was not statistically significant (*β* =–0.07, SE = 0.11, *p* =.488). Consequently, paternal pre-adoptive reflective functioning did not significantly moderate the association of child LFA at placement and total socioemotional child difficulties (*R*^2^ change = 0.01, *F*(1, 37) = 0.49, *p* =.488).

When investigating internalizing and externalizing child difficulties as perceived by adoptive fathers separately, the findings remained unchanged. LFA at placement did not significantly predict internalizing difficulties (*β* = 0.07, SE = 0.11, *p* =.512) or externalizing difficulties (*β* =–0.05, SE = 0.10, *p* =.647). Furthermore, paternal pre-adoptive reflective functioning did not directly predict internalizing difficulties (*β* = 0.07, SE = 0.11, *p* =.525) or externalizing difficulties (*β* =–0.09, SE = 0.24, *p* =.703). Similarly, the interaction term between child LFA at placement and paternal pre-adoptive reflective functioning did not significantly predict internalizing difficulties (*β* =–0.09, SE = 0.11, *p* =.456) or externalizing difficulties (*β* = 0.00, SE = 0.10, *p* =.976).

Among adoptive mothers, the total model explained a significant proportion of the variance in total socioemotional child difficulties as perceived by adoptive mothers (*R*^*2*^ = 0.42, *F*(10, 37) = 2.68, *p* =.014). Neither child LFA at placement (*β* = 0.01, SE = 0.12, *p* =.912) nor maternal pre-adoptive reflective functioning (*β* =–0.16, SE = 0.33, *p* =.622) significantly predicted total socioemotional child difficulties as perceived by mothers. Likewise, the interaction term between child LFA at placement and maternal pre-adoptive reflective functioning (*β* =–0.02, SE = 0.16, *p* =.887) was not statistically significant. Consequently, maternal pre-adoptive reflective functioning did not significantly moderate the association between child LFA at placement and total socioemotional child difficulties (*R*^2^ change = 0.00, *F*(1, 37) = 0.02, *p* =.887).

When examining internalizing and externalizing child difficulties as perceived by adoptive mothers separately, the results showed that LFA at placement was not a significant predictor of internalizing difficulties (*β* = 0.06, SE = 0.13, *p* =.610) or externalizing difficulties (*β* =–0.04, SE = 0.13, *p* =.762). Furthermore, maternal pre-adoptive reflective functioning did not directly predict internalizing difficulties (*β* =–0.11, SE = 0.35, *p* =.759) or externalizing difficulties (*β* =–0.06, SE = 0.36, *p* =.863). Finally, the interaction between child LFA at placement and maternal pre-adoptive reflective functioning did not significantly predict internalizing difficulties (*β* = 0.02, SE = 0.17, *p* =.898) or externalizing difficulties (*β* =–0.03, SE = 0.18, *p* =.867).

## Discussion and conclusions

Early adversity is widely acknowledged as a key factor influencing socioemotional outcomes, with numerous studies exploring how its severity and duration affect the adjustment of transnational adoptees. However, research in the field of transnational adoption is frequently hampered by the limited availability of detailed information about children’s pre-adoptive experiences. Furthermore, the relationship between early adversity and increased risk for socioemotional difficulties is not absolute, highlighting the need for research into sources of resilience. Identifying such factors could help buffer the effects of early adversity and inform interventions to support adoptive families.

In line with meta-analytic evidence [63], results indicated that older child age at placement was associated with more child total socioemotional difficulties 4 years after placement, controlling for the effect of BMI, LFA, child temperament, child sex, and parent education among both adoptive mothers and fathers. Although we do not discount accurate information about the children’s experiences pre-adoption, the increased risk for socioemotional difficulties associated with child age at placement has typically been attributed to prolonged exposure to early negative experiences before adoption [64]. However, as stated earlier, solely relying on child age at placement is problematic. Although studies have reported robust findings using child age at placement, its main shortcoming is that it does not capture the exposure of children to early adverse situations, nor does it take the presence of protective factors or variations in the quality of pre-adoptive care into account [65]. This underscores the need for research that examines additional factors to gain a more fine-grained perspective on early adversity.

Regarding anthropometric measures, low BMI at placement was significantly associated with total child difficulties as reported by adoptive fathers, but not by adoptive mothers, after controlling for other study variables. This association was also robust for father-reported internalizing and externalizing difficulties, suggesting that BMI at placement may capture a unique aspect of early adversity, particularly among adoptive fathers. The lack of significant associations for adoptive mothers was somewhat surprising, but may be due to a wider range in fathers’ perceptions of their child’s difficulties. Although Levene’s tests showed no significant differences between mothers and fathers in variance for internalizing, externalizing, or total socioemotional difficulties, descriptive statistics indicated that fathers tend to report a wider range of difficulties. This greater variability in fathers’ reports may increase the likelihood of detecting significant associations between BMI and socioemotional difficulties. Possibly, growth, as assessed by BMI, may reflect a combination of prenatal risk factors (e.g., maternal poverty, substance abuse, and domestic violence) and pre-adoptive risk factors (including poor nutrition, neglect, and inadequate medical care) [7, 66]. These factors could affect children’s physical growth either directly or indirectly, for example, through feeding difficulties [67]. Additionally, the socioemotional environment plays a crucial role in growth. For example, a study in Greece found that institutionalized children showed growth delays compared with family-reared children, despite being given adequate food [68]. This observation highlights the importance of a nurturing environment in physical development, which is also supported by meta-analytic evidence indicating that most children tend to catch up in the first years after adoption in terms of their growth delays [27]. Nonetheless, anthropometric measures might still shed light on the presence of early adversity in the early lives of transnationally adopted children.

Finally, consistent with research on non-adopted children, this study found that pre-adoptive reflective functioning moderated the impact of early adversity, measured using BMI as a proxy, on children’s socioemotional outcomes. Notably, this buffering effect was observed only in adoptive fathers, while its absence in adoptive mothers may reflect a statistical ceiling effect. Specifically, all adoptive mothers scored above the “borderline” score of 4 on the Reflective Functioning Scale and approximately 65% of adoptive mothers demonstrated “ordinary” or higher levels of reflective functioning, compared with 46% of adoptive fathers, most likely because of the extensive screening procedure for prospective adoptive parents in Belgium, which focuses to a large extent on the (reflective) capacities of prospective mothers of an adopted child. However, this explanation is post hoc, and there might be other explanations. There is, for example, robust evidence for a bias for social information among women [69, 70], and studies have also found that mothers use more emotion-related words than fathers when talking to their child [71, 72]. The tendency of mothers to use more emotion-related words could also have contributed to the differences between adoptive mothers and fathers, because the procedure for scoring reflective functioning typically focuses on these words in the narrative. However, we are far from completely understanding these differences, and more research is needed.

With regard to the difference between adoptive mothers and fathers in terms of their reflective functioning scores, findings are in line with other studies that suggest that women generally score higher than men on measures of mentalizing (e.g., emotion recognition, empathy, social sensitivity), a finding that is likely to be the result of biological, social, and cultural factors [48, 73]. Regarding biological factors, two recent review articles found evidence of women showing greater interest in and an attentional bias for social information compared with men, related to neurobehavioral sex differences [69, 70]. Research findings also indicate that even under stress, women seem more likely to take a social perspective and react to stress with tending and befriending behavior (i.e., predicting and identifying another’s thoughts and emotions and responding appropriately) rather than fight/flight [74]. Furthermore, socialization strategies, often rooted in culture, are likely to play a role in sex differences. Studies about sex differences in parent–child emotion narratives and parent–child play, for example, found that mothers overall used more emotion words than fathers when talking to their toddler, but that both mothers and fathers used more emotion words when talking to a daughter than to a son [71]. In addition, during play episodes, mothers appeared to be more directed toward verbally elaborating on emotions in the play of their toddler, compared with fathers [72]. However, in addition to these differences between parents, considerable within-person differences may also exist.

Taken together, findings from this study support the emphasis in the The Hague Convention [75] and the Convention on the Rights of the Child [76], which both argue that the pre-adoption history of adopted children should be documented in as much detail as possible and that each child should have the right to access this information. Furthermore, this study suggests that interventions aimed at improving the reflective capacities of adoptive parents such as Family Minds, Adopting Minds, and the Nurturing Attachments Parenting Program [31, 83, 84] might have an important role in supporting adoptive parents, particularly those parents with adopted children with high levels of early adversity. Similarly, recent systematic reviews and a meta-analysis have provided evidence for the effectiveness of other intervention programs [11, 77, 78]. The Attachment and Biobehavioral Catch-up (ABC) program, for example, is a well-established intervention for adoptive parents that has been shown to improve parental sensitivity and positive regard, reduce parental intrusiveness, foster social-emotional child competence, and decrease behavioral difficulties in children [79–81]. Future studies should further investigate the putative mechanisms of change in interventions aimed at adoptive parents generally, and the specificity of parental reflective functioning as a buffering factor and its interplay with other factors, such as parental sensitivity, in the adjustment of adoptees more specifically.

The present study has a number of strengths and limitations. One of its strengths is that it is the first study to longitudinally investigate the effect of anthropometric proxies of early adversity on child socioemotional difficulties in the context of transnational adoption, while also investigating the moderating role of pre-adoptive reflective functioning. Another strength is that the study included both mothers and fathers, consistent with the finding that the involvement of fathers is crucial in research because, like mothers, fathers play a unique role in child development [82]. Finally, the topic of transnational adoption highlights the importance of using growth standards that account for the diversity of children’s backgrounds. In this study, we used the WHO Anthro Software [83], which was developed using pooled data from six countries: Brazil, Ghana, India, Norway, Oman, and the USA. This approach ensured that the norms address variations in ethnicity as well as differences associated with low-income contexts, providing a robust framework for assessing growth across diverse populations.

There are also a number of limitations, such as the relatively small sample size, which puts the results at risk for low statistical power, especially for detecting small effects. Despite the limitations in sample size and power, our findings gain credibility through their alignment with meta-analytic evidence [20, 27] showing large effects of child age at placement and physical growth in the socioemotional development of transnationally adopted children with a history of institutionalization in orphanages and group homes. Although the relatively small sample size in this study may limit its statistical power, both the size of associations and their robustness across different studies are in line with other findings in the literature, and thus lend some confidence to their robustness. Nevertheless, future research with larger samples is needed to further investigate potential associations between early adversity and socioemotional development in transnationally adopted children.

Another limitation is the low rates of reported socioemotional difficulties, which limit the generalizability of the findings. Although these low rates could reflect positive adjustment and resilience in transnational adoptees during the early years post-adoption, several factors may have influenced these results. For example, underreporting due to social desirability bias cannot be ruled out, especially since adoption is often viewed as a positive intervention. This may also apply to other parent-reported variables in the study, such as child temperament. Additionally, difficulties that may not be immediately evident could emerge at later developmental stages, such as adolescence [84], which this study was not designed to capture. To address these issues, future research should consider using more comprehensive, multi-informant assessments that include not only parent reports but also input from teachers and the children themselves, at a later stage. Longitudinal studies that investigate the socioemotional adjustment of transnational adoptees over time would therefore be particularly valuable in identifying potential difficulties that may arise later, thereby offering a more complete understanding of how adoptive children’s socioemotional outcomes evolve.

Another important limitation is that well-validated Flemish norms are still lacking and that the use of US norms could lead to an underestimation of clinical scores [85]. Furthermore, the anthropometric measures used in this study can be seen as only a proxy of early adversity that is, moreover, based on parent-reported data. Studies using more direct measures of early adversity are needed to replicate these findings.

This study also highlights the need to examine factors beyond pre-adoptive environments, such as parent-related factors. By focusing on reflective functioning prior to adoption, the present study contributes to understanding how parental capacities measured before adoption may influence children’s socioemotional outcomes and interact with early adversity. However, reflective functioning is a dynamic construct [37] that can evolve in response to the challenges and demands adoptive parents encounter after adoption. Adoptive parents often face heightened stress, stigma, and contextual pressures [86–88], which may affect their reflective capacities. Studies have amply shown that reflective functioning has both trait and state features [89]. This study did not account for these dynamic processes, underscoring the need for longitudinal research to assess reflective functioning at multiple time points, both pre- and post-adoption. Such research would provide valuable insights into how reflective functioning adapts to the unique experiences of adoptive parenting and its role in shaping parent–child dynamics and child outcomes.

Finally, this study focused on the first 4 years post-adoption, whereas socioemotional difficulties may emerge beyond this period. Additionally, the assessment of anthropometric measures was limited to the time of placement. To gain a fuller understanding of long-term outcomes, future research should track children’s growth trajectories over several years after adoption. Such investigations could reveal important patterns of growth catch-up and their relationship to socioemotional adjustment, contributing to a more comprehensive understanding of the outcomes of transnational adoption.

Despite these limitations, this study suggests that early adversity, assessed in terms of child age and BMI at placement, was predictive of child socioemotional functioning 4 years after placement. Furthermore, pre-adoptive reflective functioning of adoptive fathers, but not of adoptive mothers, moderated the effect of early adversity on child socioemotional difficulties.

## Electronic supplementary material

Below is the link to the electronic supplementary material.

Supplementary Material 1

## Data Availability

No datasets were generated or analysed during the current study.

## Abbreviations

PA-RF: Pre-adoptive reflective functioning
BMI: Body mass index
WFA: Weight for age
LFA: Length for age
