# Supplementary material for: The moderating role of pre-adoptive reflective functioning in the association between early adversity and child difficulties after transnational adoption: a 4-year follow-up study
Source: Eur Child Adolesc Psychiatry. 2025 Jun 30;34(12):3937–53. doi: 10.1007/s00787-025-02782-x (PMC12743091; doi:10.1007/s00787-025-02782-x)
Supplement: Supplementary file 1 — Supplementary Material 1 [file 787_2025_2782_MOESM1_ESM.docx]

Supplementary Materials

European Child & Adolescent Psychiatry

The Moderating Role of Pre-Adoptive Reflective Functioning in the Association between Early Adversity and Child Difficulties after International Adoption: A 4-Year Follow-Up Study

Simon Fiore^1,2^, Nicole Vliegen^1^, Bart Soenens^2^, and Patrick Luyten^*1,3^

^1^ Faculty of Psychology and Educational Sciences, KU Leuven, Leuven, Belgium

^2^ Department of Developmental, Personality and Social Psychology, Ghent University, Ghent, Belgium

^3^Research Department of Clinical, Educational and Health Psychology, University College London, London, United Kingdom

Author Note

Simon Fiore: <https://orcid.org/0000-0001-9822-4923>

Nicole Vliegen: <https://orcid.org/0000-0002-3298-0416>

Bart Soenens: <https://orcid.org/0000-0003-1581-3656>

Patrick Luyten: <https://orcid.org/0000-0002-1161-2817>

^*^Corresponding Author: [simon.fiore@kuleuven.be](mailto:simon.fiore@kuleuven.be)

| **Table S1**  Highest Self-Reported Obtained Degree for Mothers and Fathers Separately | | |
| --- | --- | --- |
|  | **Mothers** | **Fathers** |
| High school | 12.5% | 29.17% |
| Bachelor’s degree | 47.92% | 35.72% |
| Master’s degree | 39.58% | 35.11% |
| *Note*. N = 48 couples. | | |

| **Table S2**  Child LFA at Placement as Predictor of Total Child Socioemotional Difficulties | | | | | | |
| --- | --- | --- | --- | --- | --- | --- |
|  | **Mothers** | | | **Fathers** | | |
|  | **β (*SE)*** | **[95% CI]** | ***p*-value** | **β (*SE*)** | **[95% CI]** | ***p*-value** |
| Main Effects |  |  |  |  |  |  |
| WFA 🡪 Y | –.132 (.098) | [–.331; .067] | .185 | –.091 (.085) | [–.265; .082] | .293 |
| PA-RF 🡪 Y | –.045 (.165) | [–.381; .291] | .786 | –.041 (.133) | [–.312; .230] | .759 |
| Covariates |  |  |  |  |  |  |
| CAAP | .376 (.175) | [.021; .734] | .039^*^ | .645 (.153) | [.335; .955] | .0002^***^ |
| LFA | .029 (.110) | [–.194; .253] | .792 | .051 (.093) | [–.139; .240] | .592 |
| SU | –.212 (.195) | [–.609; .185] | .285 | –.055 (.119) | [–.296; .187] | .649 |
| NA | .119 (.169) | [–.226; .463] | .489 | .112 (.145) | [–.184; .407] | .447 |
| EC | –.190 (.239) | [–.675; .295] | .432 | .142 (.173) | [–.209; .494] | .416 |
| Child Sex | –.143 (.170) | [–.489; .204] | .409 | –.215 (.128) | [–.475; .045] | .102 |
| Parent Education | .181 (.188) | [–.200; .562] | .341 | .301 (.138) | [.020; .582] | .037^*^ |
| Interaction Effect: |  |  |  |  |  |  |
| WFA x PA-RF | .066 (.094) | [–.126; .257] | .490 | .256 (.080) | [.094; .419] | .003^**^ |
| Conditional Effects : |  |  |  |  |  |  |
| Low PA-RF | – | – | – | –.444 (.124) | [–.700; –.192] | .001^***^ |
| Medium PA-RF | – | – | – | –.210 (.085) | [–.383; –.036] | .019^*^ |
| High PA-RF | – | – | – | .905 (.141) | [–.110; .391] | .262 |
| Note. † p < .10. * p < .05. ** p < .01. *** p < .001. PA-RF = Pre-Adoptive Reflective Functioning, CAAP = Child age at placement, WFA = Weight for age, LFA = Length for age, SU = Surgency, NA = Negative Affectivity, EC = Effortful Control. The values of PA-RF correspond with low (16th percentile), middle (50th percentile), and high (85th percentile) levels. | | | | | | |

.

When examining internalizing and externalizing child difficulties as perceived by adoptive mothers and fathers separately, the results showed that WFA at placement was not a significant predictor of internalizing difficulties as perceived by adoptive mothers (*β* = –.07, SE = .10, *p* = .502) or adoptive fathers (*β* = –.00, SE = .09, *p* = .971), nor did the interaction term with maternal pre-adoptive reflective functioning significantly predicted internalizing difficulties as reported by adoptive mothers (*β* = .14, SE = .10, *p* = .141). However, the interaction with paternal pre-adoptive reflective functioning did significantly predict internalizing difficulties as reported by adoptive fathers (*β* = .31, SE = .08, *p* < .001). Consequently, paternal pre-adoptive reflective functioning significantly moderated the association between WFA at placement and internalizing child difficulties as reported by adoptive fathers (*R*^2^ change = .19, *F*(1, 34) = 14.65, *p* < .001). For externalizing difficulties, WFA at placement did not significantly predict externalizing difficulties as reported by adoptive mothers (*β* = –.14, SE = .10, *p* = .176) or adoptive fathers (*β* = –.05, SE = .09, *p* = .598). Furthermore, the interaction between WFA at placement and maternal pre-adoptive reflective functioning did not predict externalizing difficulties (*β* = .05, SE = .10, *p* = .647), whereas the interaction with paternal pre-adoptive reflective functioning did significantly predict externalizing difficulties (*β* = .24, SE = ..08, *p* = .004), thereby significantly moderating the association between WFA at placement and externalizing difficulties 4 years after transnational adoption (*R*^2^ change = .11, *F*(1, 34) = 9.31, *p* = .004).

Figure 3

*Paternal Reflective Functioning, Child Age at Placement, and Internalizing Difficulties 4 Years after Placement*


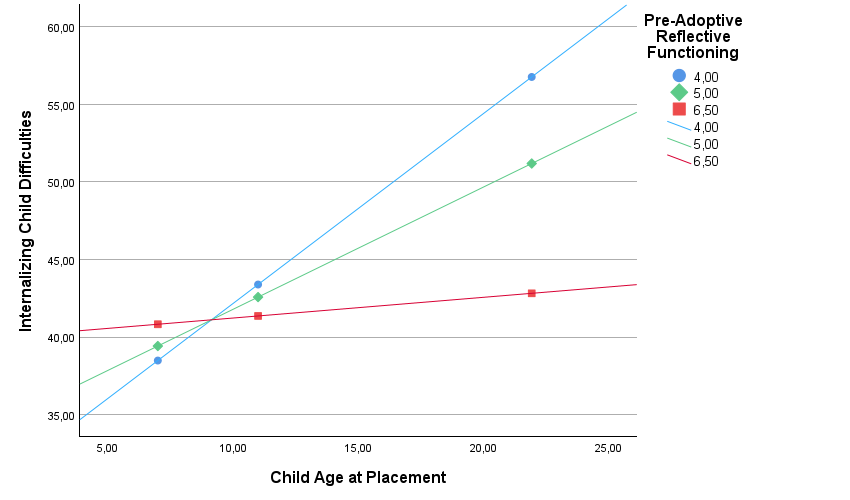


*Note.* The values of PA-RF correspond with low (16th percentile), middle (50th percentile), and high (85th percentile) levels.

Figure 4

*Paternal Reflective Functioning, Child Age at Placement, and Externalizing Difficulties 4 Years after Placement*


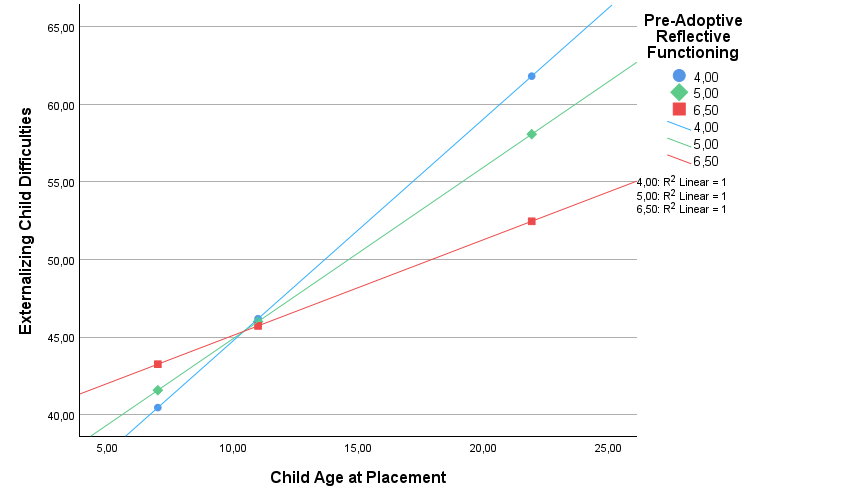


*Note.* The values of PA-RF correspond with low (16th percentile), middle (50th percentile), and high (85th percentile) levels.

Figure 5

*Paternal Reflective Functioning, Child BMI at Placement, and Internalizing Difficulties 4 Years after Placement*


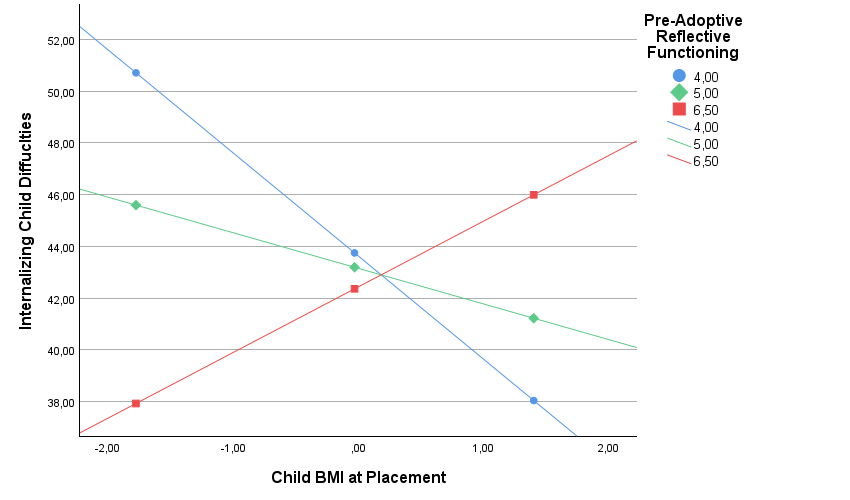


*Note.* The values of PA-RF correspond with low (16th percentile), middle (50th percentile), and high (85th percentile) levels.

Figure 6

*Paternal Reflective Functioning, Child BMI at Placement, and Externalizing Difficulties 4 Years after Placement*


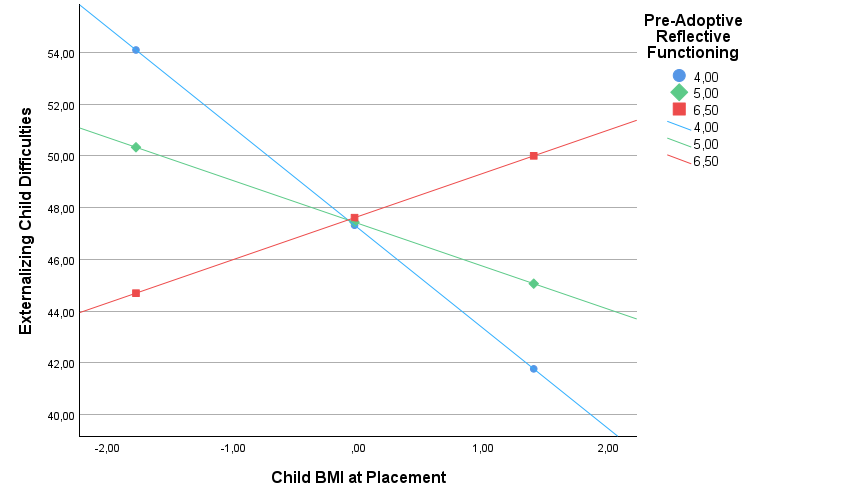


*Note.* The values of PA-RF correspond with low (16th percentile), middle (50th percentile), and high (85th percentile) levels.
